# A Novel FTIR-Based Chemometric Solution for the Assessment of Saffron Adulteration with Non-Fresh Stigmas

**DOI:** 10.3390/molecules28010033

**Published:** 2022-12-21

**Authors:** Martina Foschi, Ludovica Tozzi, Francesca Di Donato, Alessandra Biancolillo, Angelo Antonio D’Archivio

**Affiliations:** Department of Physical and Chemical Sciences, University of L’Aquila, Via Vetoio, 67100 Coppito, L’Aquila, Italy

**Keywords:** saffron, adulteration, infrared spectroscopy, chemometrics, PLS-DA, expired spice, spice

## Abstract

The development of fast, non-destructive, and green methods with adequate sensitivity for saffron authentication has important implications in the quality control of the entire production chain of this precious spice. In this context, the highly suitable sensitivity of a spectroscopic method coupled with chemometrics was verified. A total number of 334 samples were analyzed using attenuated-total-reflectance Fourier-transform infrared (ATR-FTIR) spectroscopy; the collected spectra were processed by partial-least-squares discriminant analysis (PLS-DA) to evaluate the feasibility of this study for the discrimination between compliant saffron (fresh samples produced in 2020) and saffron samples adulterated with non-fresh stigmas produced in 2018 and 2016. PLS-DA was able to classify the saffron samples in accordance with the aging time and to discriminate fresh samples from the samples adulterated with non-fresh (legally expired) stigmas, achieving 100% of both sensitivity and specificity in external prediction. Moreover, PLS regression was able to predict the adulteration level with sufficient accuracy (the root-mean-square error of prediction was approximately 3–5%). In summary, ATR-FTIR and chemometrics can be employed to highlight the illegal blending of fresh saffron with unsold stocks of expired saffron, which may be a common fraudulent practice not yet considered in the scientific literature.

## 1. Introduction

Saffron is a well-known and valuable spice obtained by drying *Crocus Sativus* Linnaeus stigmas. Nowadays, there is a great interest in this product, which is highly appreciated in the food field for its coloring and flavoring properties. This is demonstrated by the expansion of production beyond the borders of the traditional producing countries (such as Greece, India, Iran, Italy, and Spain) and by the considerable scientific literature concerning it [1,2,3]. Indeed, the saffron production process, authentication, and quality control remain topical, due to the growing consumer demand alongside the interesting biological activities and multiple applications of the spice’s phytochemicals [4]. However, saffron production still consists of several non-automated and time-consuming harvesting and post-harvesting phases that, in most cases, are conducted using traditional methods, depending on the input resources of the producing country [5]. Such condition makes saffron the most expensive spice in the world (up to 25,000 EUR/kg) and consequently a potential target of counterfeiting for profit [6].

In international trade, saffron quality is evaluated according to the ISO 3632 normative [7,8] that guarantees and approves quality control methodologies for grading saffron from all over the world.

A lower saffron quality can be due to several factors, such as inappropriate harvesting methods, insufficient dehydration processing, exposure to direct sunlight, improper storage, and, of course, adulteration [9]. In the present work, both aging and adulteration factors are addressed.

There are many studies in the literature aimed at identifying the proper analytical methodologies for the detection of molecular markers related to fraudulent practices regarding saffron; among these, headspace-solid-phase microextraction (HS-SPME) coupled with gas chromatography with mass spectrometry detection (GC-MS) proved to be an efficient method, especially when combined with chemometrics, to detect the most common plant contaminants (turmeric, calendula, and safflower) at very low levels [10,11]. In addition, electronic nose [12], ^1^H nuclear magnetic resonance [13], high-performance liquid chromatography coupled with mass spectrometry or photometric detection [14], and several methods based on polymerase chain reaction (PCR), Refs. [15,16] have been proposed. In recent years, the advantages of the application of spectroscopic techniques are driving researchers to implement untargeted but efficient methods for food and saffron authentication and quality control. Spectroscopic fingerprinting, indeed, offers simple, cheap, green, and potentially non-destructive strategies for the routine analysis of precious food matrices [17,18,19]. Concerning saffron, Chen et al., performed a feasibility study to employ infrared spectroscopy imaging and 2D correlation spectroscopy to detect plant-based adulteration [20]. Oduardi et al. presented a multi-technical approach, including spectroscopic analysis, to perform an untargeted investigation on commercial saffron samples [21]. In the context of untargeted spectroscopic approaches, specific chemometric methods, such as multivariate curve resolution—alternating least-squares (MCR-ALS), can usefully support the identification and quantification of unknown adulterants. For instance, Castro and co-workers demonstrated that near infrared spectroscopy coupled with MRC-ALS can identify and quantify several contaminants in saffron samples at very low levels [22]. Fourier-transform mid-infrared (FTIR) spectroscopy and pattern-recognition methods were also used to discriminate authentic saffron from colorant-added saffron and adulterated samples with common plant-based contaminants [23,24,25]. Diffuse reflectance FTIR spectroscopy was in particular employed to detect six different common saffron adulterants of plant origin, i.e., *Crocus sativus* stamens, calendula, safflower, turmeric, buddleja, and gardenia, at relatively low contamination levels (from 5 to 20% *w/w*) [26]. 

Although the most common adulteration practices consist in the addition of artificial or natural dyes (tartrazine, gardenia yellow, extract of *Buddleja officinalis* M.) or parts of cheaper plants (safflower, calendula, turmeric), it is not unrealistic that dishonest producers could mix fresh saffron with unsold aged stocks produced in previous years. Unfortunately, the latter is an auto-adulteration practice that is difficult to reveal on both powdered and intact stigmas, at least through visual inspection or microscopic analysis, which is the method suggested by the ISO 3632 procedure to detect extraneous materials. On the other hand, as Sabatino et al. [14] reported, the UV-Vis spectroscopic analysis of aqueous extracts, proposed by the ISO-3632 normative for grading saffron, also seems inappropriate for revealing the addition of expired saffron to the fresh spice. In this context, the effect of aging on the content of specific saffron metabolites has been described in the literature [27,28,29]. Consonni and co-workers, in particular, investigated aging effects on the saffron composition by the application of either ^1^H nuclear magnetic resonance [28] and infrared spectroscopy [30]. These authors classify as “fresh” the saffron samples stored for up to four years and as “non-fresh” the saffron samples stored for more than four years, based on the evidence that within four years from production the changes in the relative content of molecular markers of quality deterioration (sugars bound to crocetin, glucose in picrocrocin, free sugars and fatty acids) are negligible. Nevertheless, a best-before date of two years is established for saffron marketed in Italy, to preserve the original quality of the spice. It follows that the class of fresh samples, as conceived by Consonni and co-workers, actually includes a number of legally expired products unsuitable to be placed on the market.

In light of the evidence in the literature and the legal expiration time of two years established for saffron marketing, we evaluated in the present work the sensitivity of ATR-FTIR spectroscopy in recognizing saffron adulterated with legally expired stigma and quantifying the contamination level. To achieve these goals, in 2021 we collected the infrared spectra of saffron samples produced in 2020, taken as compliant (fresh) products, and re-analysed saffron samples produced in 2016 and 2018, together with mixtures of fresh stigmas (2020) and aged (2016 or 2018) stigmas, to simulate adulteration of the genuine spice with expired products. A sufficiently great number of ATR-FTIR spectra were handled by partial-least-squares discriminant analysis (PLS-DA) to attempt a classification of the saffron samples according to the aging time and discrimination between fresh samples and samples adulterated with expired products. In addition, PLS regression was applied to estimate the quantitative level of such kind of adulteration. Based on our best knowledge of the literature, no other studies have been carried out before on this subject. 

## 2. Results and Discussion

**Figure 1:**
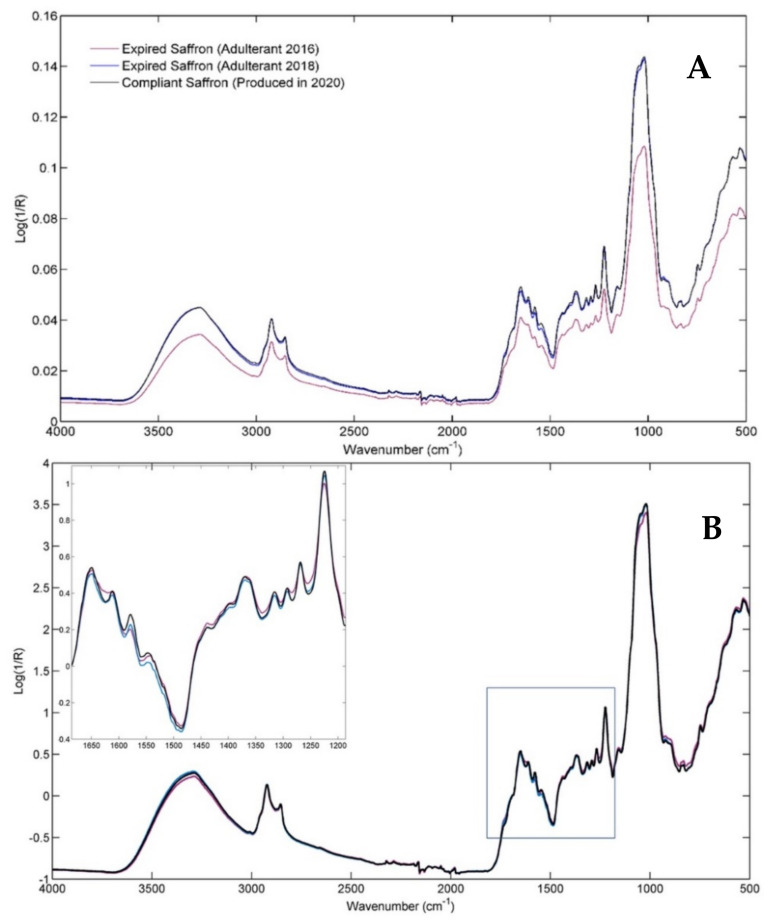
(**A**) Mean row infrared spectra for the Compliant class (in black) and for the two considered pure Adulterants, i.e., Adulterant 2016 (in purple) and Adulterant 2018 (in blue). (**B**) Mean signals for the SNV-pre-processed spectra, distinguished again according to the three classes.

Figure 1 shows the ATR-FTIR mean spectra of powdered saffron samples, averaged according to the production year, and hence according to the aging time. At the moment of the analysis (2021), saffron produced in 2020 could still be marketed, whereas the spices produced in 2018 and 2016 are no longer compliant, according to the legal expiration time of two years from production.

Since a visible instrumental shift can be confirmed by a simple visual inspection of the mean spectra, standard normal variate (SNV) pre-treatment was applied to the raw data to obtain corrected signals reported in Figure 1B. As a result, a minimal signal-intensities change is recognizable on all the major peaks of the three spectroscopic profiles. However, different intensity ratios of the spectral variables can be revealed, especially concerning the fingerprint range of the saffron samples produced in 2016, namely expired for three years (see the detail in Figure 1B). A summary interpretation of the major infrared bands is provided below, in which the attribution of the observed signals to the main saffron components was achieved by referring to the literature [29,31].

The broad band centered at approximately 3300 cm^−1^ is related to the bonded and not-bonded hydroxyl (O-H) stretching, while the peaks at 2924 cm^−1^ and 2854 cm^−1^ correspond to C-H asymmetric and symmetric stretching vibrations. By moving into the fingerprint region, the two shoulders centered at 1745 cm^−1^ and 1710 cm^−1^ are attributed to the -C=O stretching in crocins, free carboxylic groups of crocetin (a conjugated bicarboxylic acid), and aldehydes (such as picrocrocin and safranal). The band from 1655 cm^−1^ to 1645 cm^−1^ is attributed to the conjugated C=C stretching vibrations typical of carotenoids and apocarotenoids, but the amide I band and the O–H bending vibrations of water also fall in this spectral region. The second well-recognizable peak in the 1800–1500 cm^−1^ fingerprint range comes from skeletal vibrations (C-C at 1613 cm^−1^); nevertheless, the signal related to C=C vibration of α,β-unsaturated ketones can also be found in this spectral region. The peak at 1578 cm^−1^ and the shoulder at 1545 cm^−1^ are related to the C–O vibrations in conjugated esters, as well as to the amide II and aromatic C=C stretching vibrations. The combined vibrations of CH/CH_2_, OH, C-C, C-O, and CCO moieties contribute to the 1454–1221 cm^−1^ fingerprint zone. In this area, the band at 1221 cm^−1^ related to the (C=O)-O group of the crocetin esters is particularly interesting. The range from 1157 cm^−1^ to 1020 cm^−1^ is dominated by signals of the sugar units and the glycosidic linkages in polysaccharides or glycosyl moieties of crocins and flavonoids. The signal at 1157 cm^−1^ can be attributed to the C-O stretching vibration, whereas the intense bands at 1051 cm^−1^ and 1020 cm^−1^ are associated with C-O-C glycosidic linkages of oligosaccharides and to the bending vibration in sugars. The shoulder from 970 cm^−1^ to 920 cm^−1^ results from the skeletal vibration modes of the glycosidic linkages as well as the trans = C–H out-of-plane bending, which are typically of trans-crocins. Finally, the region around 740 cm^−1^ could be related to the cis = C–H out-of-plane bending, typical of the cis-isomers of crocins.

Overall, the difficulties of interpreting the differences in terms of compositional changes are evident, due to the complexity of the matrix under analysis and the spurious effects introducing variability unrelated to the chemical characteristics of the samples. Consequently, appropriate chemometric methods have been applied to exploit the spectroscopic signals and obtain helpful information about the problem under investigation.

### 2.1. Discrimination among Compliant and Differently Adulterated Saffron 

To avoid over-fitting, all the models were built on a training set consisting of 70% of the collected samples, selected employing the duplex algorithm separately for each class. The models were optimized in terms of number of latent variables (LVs) and pre-treatment of the spectra with a 5-fold cross-validation (CV) procedure, by considering the Mean correct classification rate (CCR %) in CV. First of all, a PLS-DA model was built in order to verify the chemical differences among compliant samples (saffron produced in 2020) and the two classes of expired saffron (produced both in 2018 and 2016). From now on, this first model will be referred to as Model I.

#### 2.1.1. Model I

**Figure 2:**
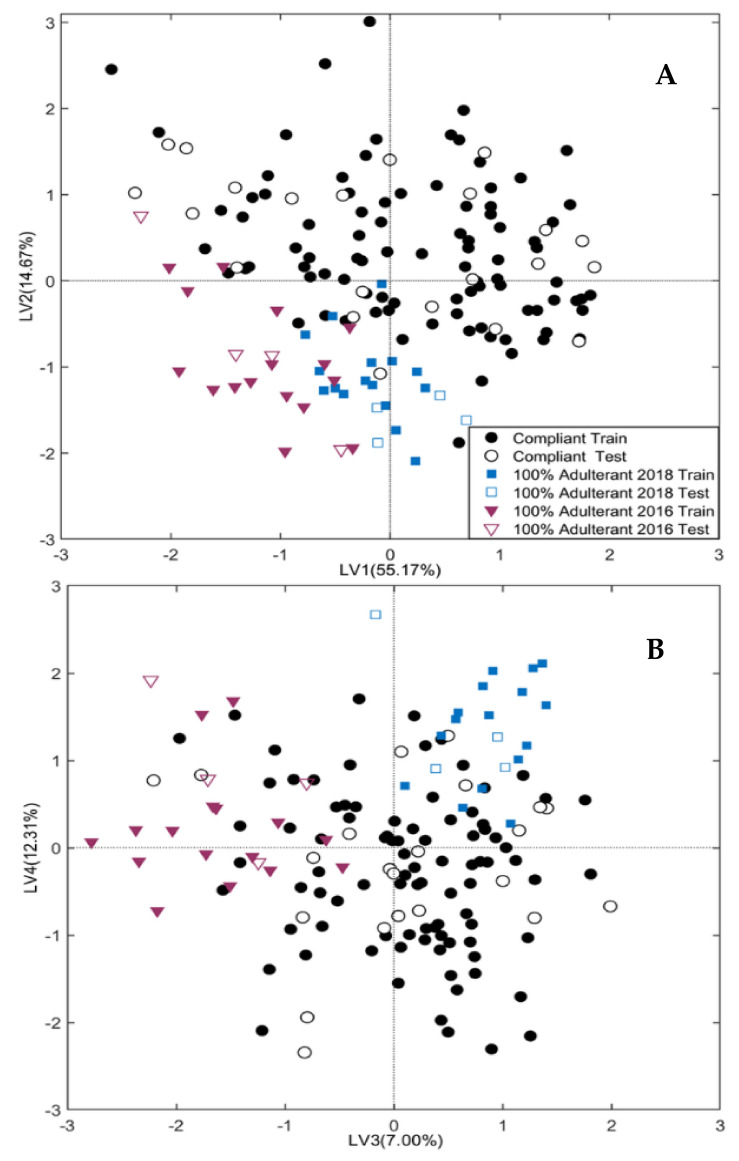
Sample scores onto the LV1-LV2 space (**A**) and LV3-LV4 space (**B**) for Model I, built on Compliant samples and Pure Adulterants (100% Adulterant 2018 and 100% Adulterant 2016).

Model I is graphically displayed in Figure 2, which reports the projection of the training (full symbols) and test (empty symbols) samples onto the four significant LVs.

Figure 2A,B, in particular, displays the projection of the samples onto the LV1-LV2 and LV3-LV4 plane, respectively. The classification rates of Model I in cross-validation and external prediction are summarized in Table 1

Although non-fresh saffron samples are poorly represented in this preliminary model, the score plots of Figure 2 show an excellent agreement between the training and test samples, resulting in a good model generalization. Moreover, the bisector of the I-III quadrant distinguishes between fresh and non-fresh samples; in detail, non-fresh samples cluster in the third quadrant of the LV1-LV2 space, falling at negative scores for both LV1 and LV2. It is interesting to note that the direction identified by the LV3 (7% of **X**-block explained variance) is able to discriminate the samples produced in 2016, which fall at negative scores, from samples produced in 2018, falling at positive scores, while most of the fresh samples (produced in 2020) are located between the above two classes collecting the expired samples. It follows that LV3 cannot be simply related to the aging time. In this regard, it must be stressed that the quality control on the saffron samples here considered, performed according to ISO-3632 procedures, within one year from their production (unpublished results) revealed an exceptionally higher quality for the samples produced in 2018. It must be concluded that LV3 not only describes the effect of aging but also encodes the variability associated with the year-dependent original composition of the saffron samples, which seems to be interconnected with the aging effects. 

Model I is capable of attributing all the test samples except three. In particular, one non-fresh sample of 2018 was erroneously attributed to the 2016 class, and the class of fresh samples erroneously refused two genuine samples. The good classification performance of Model I demonstrates that ATR-FTIR has an acceptable sensitivity for detecting the changes in the saffron composition determined by aging under domestic storage conditions (away from heat and light sources, and in airtight containers).

#### 2.1.2. Variable Importance in Projection (VIP) Analysis

Variable-importance-in-projection (VIP) coefficients express the importance of each variable in defining the LV subspace [32]; a VIP index equal to 1, which is the average of the squared VIP values, is assumed as a cut-off to define the significant variables.

**Figure 3:**
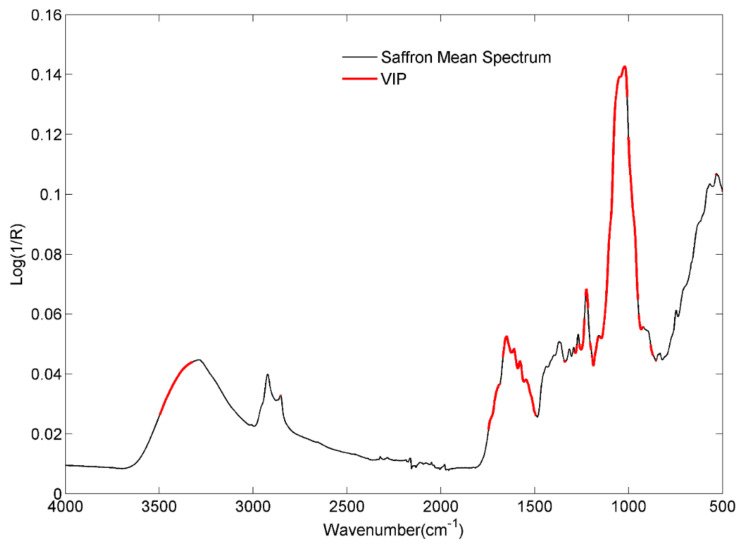
Mean saffron spectrum in black and significant spectral variables highlighted in red.

Figure 3 reports the graphical outcome of the VIP analysis related to Model I. The red-highlighted portions of the spectrum reveal the most significant spectral variables for the discrimination of the three classes, namely fresh (2020), non-fresh (2016) and non-fresh (2018). In other terms, Figure 3 shows the spectral ranges mostly influenced by the aging process, useful for differentiating the compliant (fresh) products from the legally expired ones. In this regard, a more detailed attribution of the absorption bands to correlate them with the degradation mechanisms proposed in the literature is helpful [33,34,35].

Thus, starting from the highlighted range 3322–3498 cm^−1^, this can be attributed to the moisture loss in samples. However, the oxidative degradation of double bonds, thermal degradation, and hydrolysis could lead to the formation or loss of free hydroxyl groups, affecting the signal intensity of these spectral variables. The two broad shoulders centered at 1745 cm^−1^ and 1710 cm^−1^ can be attributed to the C=O of α,β-unsaturated esters, as in the case of crocins, although falling in the same range is the C=O stretching of conjugated aldehydes, such as the picrocrocin, safranal, and HTCC (4-hydroxy-2,6,6-trimethyl-3-oxocyclohexa-1,4-diene-1-carboxaldehyde), all compounds that proved to be affected by aging.

The bands at 1650 cm^−1^ and 1610 cm^−1^ are due to C=C stretching of conjugated alkenes and α,β-unsaturated ketones; among the most important compounds in the literature, the isophorone appears to be the volatile species, together with the HTCC, which is most involved in the degradation of saffron during aging. According to Maggi and co-workers [27], HTCC and isophorone significantly decrease when moving from 1 to 3–4 years of storage. The signals at 1580 cm^−1^ and 1545 cm^−1^ are associated with the aromatic rings and cyclic alkenes, whereas the signals around 1227 cm^−1^, 1157 cm^−1^, 1051 cm^−1^, and 1028 cm^−1^ are associated with glycosidic bonds and free sugars. Finally, the shoulder at 986 cm^−1^ and the signals around 880 are related to the C=C bending, the first being related to the trans configuration of apocarotenoid pigments, which are supposed to be the main actors of saffron degradation. Furthermore, one of the degradation phenomena observed, which involves partial or total hydrolysis, due to the breaking of glycosidic bonds in crocins and picrocrocin, can explain the selection of variables between 1227 cm^−1^ and 1029 cm^−1^ [28,30].

The results of the VIP analysis were reported for the purpose of variable ranking, which is useful in assisting the interpretation of the data in this preliminary stage.

**Figure 4:**
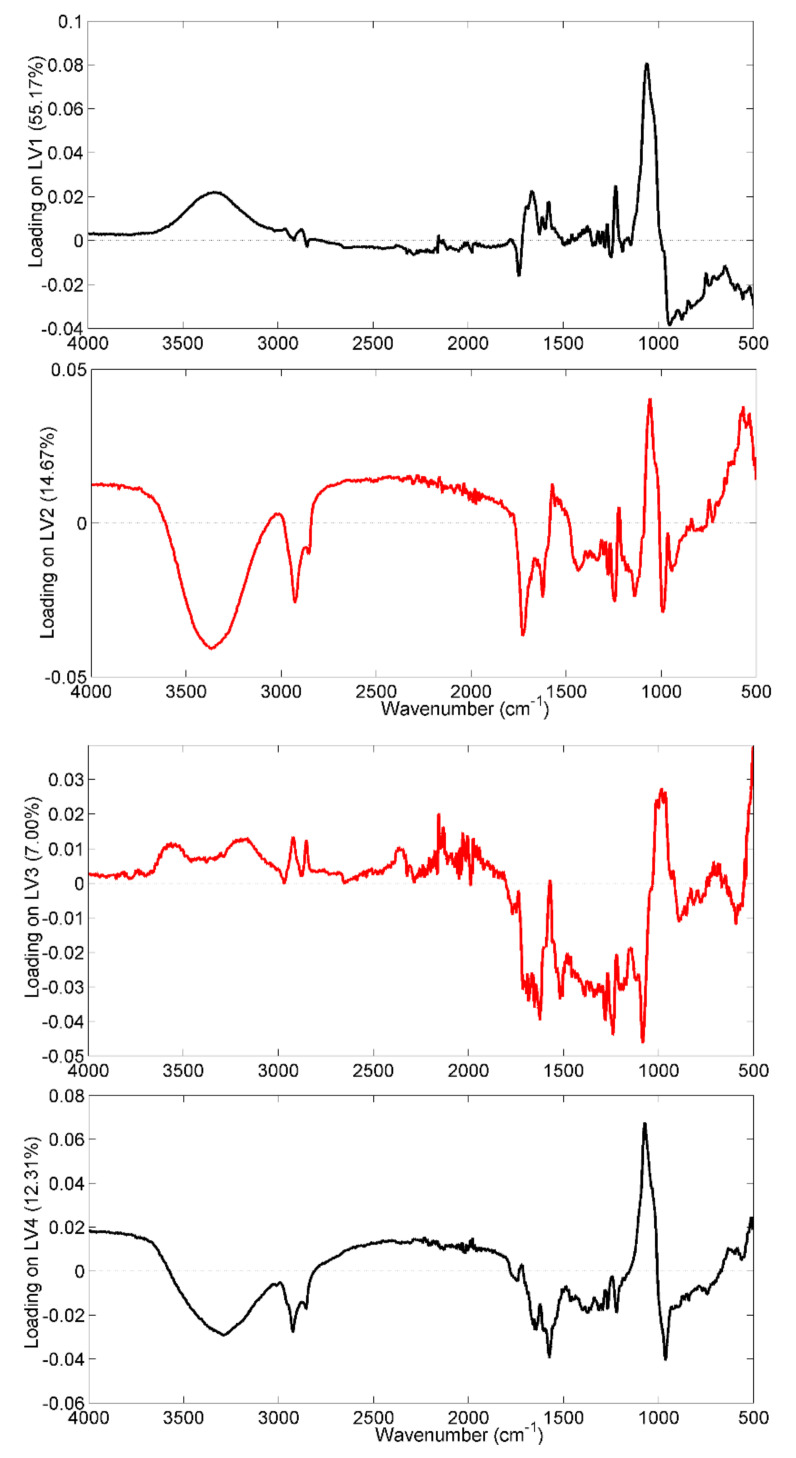
Loadings of the spectral variables on the four considered Model I LVs. Highlighted in red are the loadings on LV2 and LV3, the directions along which a trend related to spice aging and the annual microclimatic effects, respectively, could be evaluated.

However, noting the different sources of variability in the dataset, the analysis of the loading plots, related to the four latent variables, could provide some interesting information which would be useful for commenting on the effects of aging on the IR spectrum of saffron versus those related to annual microclimatic variability. In this regard, Figure 4, where loadings on all the four latent variables of Model I are plotted, is shown and commented on.

Starting from the previous considerations about the assignment of the IR absorption bands, samples produced in 2018, which fall at positive scores of the LV3, appear to be potentially rich in the *trans*-crocins portion.

Indeed, all the bands with positive loadings (the one centered at 980 cm^−1^, those centered at 3600 cm^−1^ and 3400 cm^−1^, and the two peaks around 2920 cm^−1^ and 2850 cm^−1^) showed higher intensity in the samples harvested in 2018. On the contrary, the fingerprinting zone, to which all the aldehydic compounds related to coloring and aromatic power also contribute, appears less intense for saffron samples produced in 2018. This interpretation may be in line with the particularly high coloring strength of the 2018 samples, evaluated during the UV-Vis quality control of the spice.

As regards the aging effect, the discussion was limited to the variables having concordant loadings for both the first and second latent variables, since the expired saffron samples appeared to differ along the bisector of the I-III quadrant of the space described by LV1 and LV2. In this case, it is interesting to note that the variables centered at 1057 cm^−1^ and 1570 cm^−1^, related to the glycosidic bonds between sugar residues and to the ester bonds of crocins, are more intense in the fresh samples, whereas the peak at 1730 cm^−1^, related to aldehydes and free carboxylic acids, is more intense in the case of non-fresh samples; these findings are in agreement with the hydrolytic reaction that occurs in the storage period of 0–4 years [28].

#### 2.1.3. Model II

After the construction of a preliminary discrimination model, useful for assessing the feasibility of the present study, a second PLS-DA model was built to distinguish compliant (fresh) saffron samples from samples adulterated with expired stigmas, produced in two different years (2016 and 2018) at different adulteration levels. Again, a three-class discriminant model (Model II) was constructed, although, to make the treatment more streamlined, the results are reported in terms of specificity and sensitivity for the class of fresh saffron. From now on, the three classes in the problem will be referred to as Compliant (fresh saffron produced in 2020), Adulterated 2016 (fresh saffron mixed with expired stigma produced in 2016) and Adulterated 2018 (fresh saffron mixed with expired stigma produced in 2018). As described in Section 3.1, the Adulterated 2016 and Adulterated 2018 classes are made of three adulteration levels (from 40% to 10%) and pure expired stigmas produced in 2016 and 2018. Following the method described in Reference [36], a probabilistic binary method was employed as a classification criterion. Referring to the responses calculated by the three-class model, for each column of the predicted response matrix in calibration (${\hat{Y}}_{\mathrm{cal}}$) two Gaussian distributions were identified (for Compliant and Not-Compliant (Adulterated 2016 + Adulterated 2018) samples, for instance). This procedure was performed for each of the three classes of the problem, allowing for the computing of the class probability-density-functions and to select a class-specific threshold within which a sample is attributed to the category. In detail, the threshold will be the response value where the two curves cross, which corresponds to 50% of the normalized probability. Thus, considering the ${\hat{y}}_{new}$, i.e., the column vector of the responses for a new sample, a posterior probability value can be calculated for each G-class. The sample will be attributed to the classes for which the posterior probabilities are greater than 50%; it will be attributed to none of the classes if their posterior probabilities do not exceed 50%. Moreover, if the i-th new sample has a ${\hat{y}}_{i,G}$> max (${\hat{y}}_{\mathrm{cal},G}$) (where ${\hat{y}}_{\mathrm{cal},G}$ is the predicted column vector in calibration for the G-th class) the posterior probability must be extrapolated, so the sample will not be attributed to the G-th class. Consequently, this method admits non-attributions or ambiguous classifications. Although this procedure is extended to all the three classes considered, the discussion is focused on the class of interest, i.e., on the Compliant samples.

**Figure 5:**
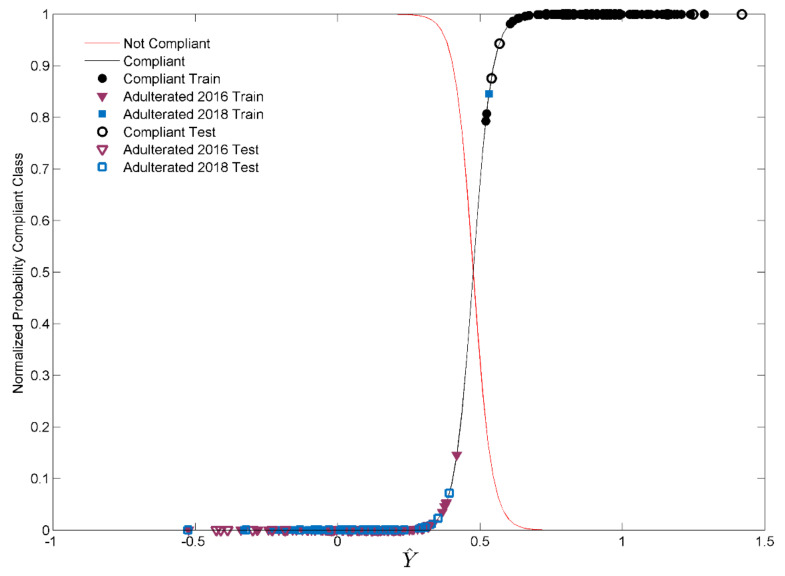
Normalized probability of the Compliant class (black curve) and posterior probability of training (full symbols) and test samples (empty symbols) for Model II.

Figure 5 reports the graphical results for Model II, built on pre-treated spectroscopic signals (standard normal variate (SNV) and mean centering (MC)) and considering an optimized number of 13 LVs. In detail, the normalized class probability for the Compliant category is reported in black, whereas the line highlighted in red represents that for all the non-compliant samples (Adulterated 2018 and Adulterated 2016). The intersection between the two curves corresponds to the 50 % probability and represents the threshold for assignment of the objects to the Compliant class. As can be seen in Figure 5, all the samples in the test set are correctly recognized by the Model II (100% sensitivity in external prediction), whereas all the non-compliant (Adulterated 2018 and Adulterated 2016) samples are rejected, resulting in a 100% specificity in external prediction. A misclassification case occurs only in calibration, in which the class of Compliant samples incorrectly accepts a sample adulterated with saffron produced in 2018.

### 2.2. Quantification of the Adulterants by Means of PLS Regression

For regression purposes, we built two subsets to be elaborated separately. The first is made of all the 112 compliant samples and all the contaminated saffron cases obtained by mixing fresh saffron with saffron produced in 2016; the second subset consists of all the compliant samples and those adulterated with saffron produced in 2018. 

The quantitative response value, related to the actual percentage of adulteration, was assigned to each sample and calculated by considering the adulterant weight over the total batch weight. The model was optimized by cross-validation (CV) on 70% of samples in the training set, identified by applying the duplex algorithm [37] separately for each adulteration level. Unlike the PLS-DA data treatment, pure saffron samples produced in 2016 and 2018 (corresponding to a 100% contamination level) were not included in the two datasets, because the extension of the respective PLS models to such a large concentration range (0–100%) resulted in an unacceptable worsening of the CV predictive performance. 

**Figure 6:**
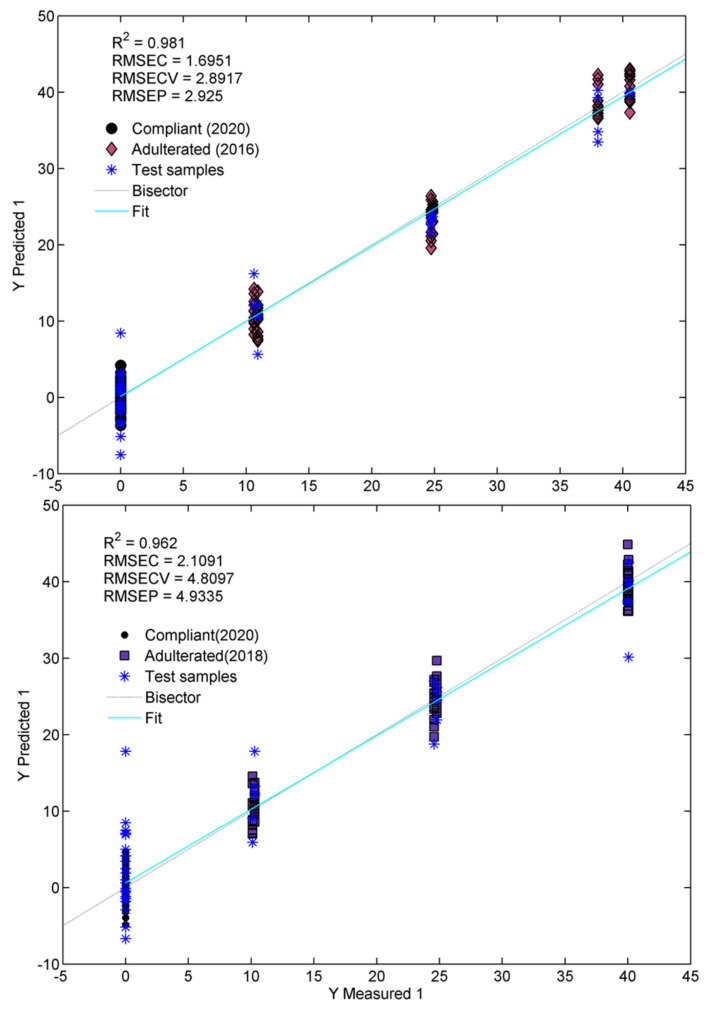
Plot of measured against predicted responses (% of adulteration) and related figure of merits.

As seen in Figure 6 and Table 2, the model obtained for saffron adulterated with non-fresh stigmas produced in 2016 shows an excellent agreement of the measured against predicted percentages of adulteration. This can be confirmed by the determination coefficient in prediction (Q^2^) value of 0.961 and by the root-mean-square error of prediction (RMSEP) of 2.92%. In the case of saffron adulterated with non-fresh products of 2018, the results are slightly worse, since we have a Q^2^ of 0.916 and an RMSEP of 4.81%. The lower aging of the Adulterant 2018 objects, compared to those of 2016, can certainly explain the above results. However, as reported in Section 2.1.1 on the trends highlighted by Model I, it is possible that the exceptionally high quality of the saffron samples produced in 2018, probably related to favorable microclimatic conditions, may have partially covered the effects of the aging phenomena on the chemical composition of these samples. In this context, moisture content is also a critical variable that should be considered when dealing with aging processes. It should be remarked that the analyzed samples probably did not undergo different extents in the deterioration processes induced by different amounts of moisture, since the original water content was always around 10%, regardless of the producer and the production year. In addition, although we did not have enough of the aged samples here analyzed (those produced in 2016 and 2018) to evaluate their moisture content at the moment of the ATR-FTIR analysis, we observed in previous saffron characterizations that the loss of water is almost negligible in the saffron stigmas stored under the same conditions as in this work (in the dark, at room temperature and in closed containers), even after three to four years after production.

## 3. Materials and Methods

### 3.1. Data Set

A total number of 334 samples, comprised of genuine and adulterated, subdivided as shown in Table 3, were analyzed using ATR-FTIR spectroscopy. The saffron samples were kindly donated by producers from the geographical area around Spoleto (20 producers) and Città della Pieve (15 producers) (Umbria region, Central Italy) some months after the production cycle was completed, in the harvesting years 2016, 2018 and 2020. In detail, some aliquots of saffron provided by the different producers in 2020 (referred to as Compliant saffron) were sampled in order to obtain five batches of “fresh” saffron. Non-compliant samples were artificially generated by mixing fresh saffron with expired products (those produced in 2016 or 2018) at three different percentages, from 10% to 45%, to obtain three batches for each adulteration level and each production year of the aged products. Therefore, considering the two-year expiration date legally assumed for saffron, the spice produced in 2016 was a 3-year-expired product, bearing in mind that the analyses were conducted in 2021. The second kind of contamination was due to the addition of saffron stigmas produced in 2018, which had expired for just one year.

### 3.2. Instrumental Analysis—ATR-FT-MIR Spectroscopy

Just before analysis, each aliquot of whole stigmas was finely ground with a mortar, homogenized, and analyzed. Approximately 22 analyses were performed on each batch of the compliant samples, while approximate y10 analyses were performed on each batch of artificially adulterated samples. The infrared spectra were collected using a PerkinElmer Spectrum Two™ (PerkinElmer, Waltham, MA, USA) FTIR spectrometer equipped with a deuterated-triglycine-sulfate detector and a PerkinElmer Universal Attenuated Total Reflectance module (uATR) with a single-bounce diamond crystal. After the background was carried out by exposing the crystal in the air, the ground saffron samples were carefully placed on the ATR module so as to cover the crystal totally, and a constant force was applied, using the pressure monitoring system integrated with the instrument. All the spectra were registered from 4000 to 500 cm^−1^ with an instrumental resolution of 4 cm^−1^ as the averages of ten scans. All the spectra were converted in the pseudo-absorbance scale (Log (1/Reflectance)), exported, and processed in MATLAB (The Mathworks, Natick, MA, USA; version 2015b) using in-house functions.

### 3.3. Chemometric Tools for Calibration and Validation

The quantification of substances in complex matrices is a problem that can be solved by means of regression methods which, starting from a set of measures X (*predictors*) and a response matrix Y (or y, if only one response is modelled), allow for the estimation of the coefficient matrix B (and the residuals matrix E, containing the unmodelled information), which regulates the relationship between X and Y, as described by the generic regression (Equation (1)):

(1) Y=XB+E

Several approaches have been proposed to solve Equation (1); in the case of interferers that make it difficult to quantify a substance of interest, and/or of correlated predictor variables (as often happens in spectroscopy), one of the most commonly used methods is partial least squares (PLS) [38,39,40]. 

Very briefly, this is made possible by the decomposition of X and Y into a set of X- and Y-scores (**T** and **U**, respectively), of X- and Y-loadings (**P** and **Q**, respectively), and by the estimation of the matrix **C,** which adjusts their *inner relation*. Overall, this allows for the estimation of ***B***, and thus the creation of a calibration model which can be used for the quantification of the compound of interest (i.e., the estimation of the response ${\hat{Y}}_{new}$) in new samples:

(2) $${\hat{Y}}_{new}=X_{new}B+E$$

PLS has a large number of benefits over other regression approaches [41]. Furthermore, it has also been exploited for the realization of a classification method called partial-least-squares discriminant analysis (PLS-DA) [42]. This approach is based on the assumption that a classification problem can be transformed into a regression one and solved by PLS. This is enabled by a suitable codification of class membership, achieved by the so-called Y-dummy. This response matrix is a binary array encoding the class-belonging of each sample. For instance, in a three-class problem, a sample appertaining to class 1 is represented by a y vector **y_1_** = [1 0 0], an object belonging to class 2 is represented by the vector **y_2_** = [0 1 0] and a sample from class 3 is codified as **y_3_** = [0 0 1]. Once the Y dummy is created, the calibration model is achieved by solving Equation (1) using PLS. In the case of the prediction of class-membership of a new sample being necessary, this can be obtained by solving Equation (2). Nevertheless, ${\hat{Y}}_{new}$ is no longer a categorical matrix but it is constituted of continuous values, so the assessment of the class-membership of new samples is not straightforward. Different approaches have been proposed in this regard; here, the solution proposed by Perez et al. described in [43] has been used.

## 4. Conclusions

An adulteration practice not yet addressed in the literature was reported and evaluated in this work. In this context, both aging and adulteration factors were addressed as the main effects affecting the commercial quality of saffron. The strength of the ATR-FTIR fingerprinting method combined with chemometrics (PLS-based classification and regression) was verified. Through the application of a fast, cheap, and non-destructive method, the freshness of saffron was verified and the samples were classified by means of PLS-DA, in accordance with the aging time. PLS-DA was also employed to discriminate between compliant (fresh) and non-compliant (adulterated with expired products) saffron samples, achieving an excellent prediction ability. Moreover, a PLS-regression approach was used to predict with sufficient accuracy the adulteration level for two different aging times of the expired products. In conclusion, ATR-FTIR spectroscopy and chemometrics can be used to detect the illegal addition of aged saffron to fresh products or to reveal fraudulent mislabeling with respect to the best-before date.

## Figures and Tables

**Table 1:** Summary of the PLS-DA outcomes for both Model I and Model II. The optimal pre-treatment, the optimal number of latent variables (LV), the percentage of mean correct classification rate in cross-validation (CCR(%) CV), as well as the predicted specificity and sensitivity for the Compliant class (Comp. specificity; Comp sensitivity), are reported.

PLS-DA Outcomes					
	Pre-Treatment	LV	Mean CCR (%) CV	Comp. Specificity (%)	Comp. Sensitivity (%)
Model I	SNV + MC	4	100%	100%	90.5%
Model II	SNV + MC	13	99.2%	100%	100%

**Table 2:** Summary of PLS-R outcomes in terms of optimal pre-treatment (first derivative (D1), SNV, and MC), number of latent variables and root mean square error in cross -validation (RMSECV) and prediction (RMSEP).

PLS-Regression Outcomes				
	Pre-Treatment	LV	RMSECV	RMSEP
**2016** Adulterant	D1+SNV+MC	10	2.96%	2.86%
**2018** Adulterant	SNV+MC	15	4.6%	4.3%

**Table 3:** The number of analyzed samples at the different adulteration levels and divided into Adulterated and Compliant saffron samples.

	N. Adulterated Samples				N. Compliant Samples
Adulteration Level	100%	40%	25%	10%	0%
**2016** Adulterant	20	30	30	32	102
**2018** Adulterant	20	30	30	30	

## Data Availability

The data presented in this study are available on request from the corresponding author.
